# Impact of Losartan Nanoparticles on Carbon Tetrachloride-induced Cerebellar Injury in Rats: Association with Alterations in RIPK1/RIPK3/MLKL Necroptosis Markers

**DOI:** 10.1007/s12035-026-06178-z

**Published:** 2026-09-17

**Authors:** Nermeen H. Lashine, Heba M. Elhessy, Maha O. Hammad, Eman A. E. Farrag, Shereen Hamed, Ola Ali Habotta, Basma Adel Khattab

**Affiliations:** 1 Department of Human Anatomy and Embryology, Faculty of Medicine, Mansoura University, Mansoura, Egypt; 2 Department of Medical Biochemistry and Molecular Biology, Faculty of Medicine, Mansoura University, P.O.No.35516, Mansoura, Egypt; 3 Department of Clinical Pharmacology, Faculty of Medicine, Mansoura University, Mansoura, Egypt; 4 Department of Medical Histology and Cell Biology, Faculty of Medicine, Mansoura University, Mansoura, Egypt; 5 Department of Forensic and Toxicology, Faculty of Veterinary Medicine, Mansoura University, Mansoura, Egypt; 6 Department of Anatomy and Embryology, Faculty of Medicine, Damietta university, Damietta, Egypt; 7 Department of Anatomy and Histology, Faculty of Health Science Technology, Mansoura National University, Gamsa, Egypt; 8 Horus Research Center, Horus University, New Damietta, Egypt

**Keywords:** Losartan, Cerebellar toxicity, Nanoparticles, Necroptosis, CCL

## Abstract

Carbon tetrachloride (CCL₄) is a lipophilic industrial solvent metabolized by cytochrome P450 enzymes. Its rapid diffusion across membranes facilitates widespread tissue distribution, including the brain, where it induces oxidative stress and alters the expression of necroptosis-related markers, including tumor necrosis factor-alpha (TNF-α). Losartan, an angiotensin II receptor antagonist, has demonstrated attenuation of cerebellar injury in many neurodegenerative diseases. This study evaluated the potentials of losartan and its nanoparticle formulation against CCL₄-induced cerebellar injury. Twenty-four adult male Sprague–Dawley rats were assigned into four groups: Control, CCL₄, Losartan, and Losartan potassium nanoparticles (LP-NPs)-treated groups. Oxidative stress parameters, quantitative Real time- PCR (qRT-PCR) for Tnf-α, receptor interacting protein kinase 1 (Ripk1), receptor interacting protein kinase 3 (Ripk3), and mixed lineage kinase domain-like protein (Mlkl), along with histological and immunohistochemical (IHC) assessments, were performed. CCL₄ exposure significantly elevated malondialdehyde (MDA) while reducing superoxide dismutase (SOD), reduced glutathione (GSH), and catalase. Molecular study shows upregulation of mRNA expression of *Tnf-α*, *Ripk1*, *Ripk3*, and *Mlkl*, which correlates with alterations in necroptosis-related marker expression. IHC showed marked increases in RIPK1, RIPK3, Glial Fibrillary Acidic Protein (GFAP), and Ionized calcium-binding adaptor molecule1 (Iba1) immunoreactivity, with a concomitant decrease in caspase-8. Histologically, CCL₄ induced severe cerebellar alterations, including reduced myelinated fiber area, Purkinje cell loss, and granular layer thinning. Both Losartan- and LP-NPs-treated groups significantly mitigated these biochemical, molecular, and histological changes, with LP-NPs exerting greater observed effects in histological parameters and selected IHC markers. Both treatments produced comparable reductions in qRT-PCR mRNA expressions of *Tnf-*α*, Ripk1*, and *Ripk3*. In conclusion, losartan, particularly in nanoparticle form, attenuates CCL₄-induced cerebellar toxicity and is associated with alteration in necroptosis-related markers and attenuation of GFAP and Iba1 immunoreactivity, accompanied by structural improvement. These findings highlighted LP-NPs as a prospective biochemical, molecular, and histological protective agent against CCL₄-induced cerebellar injury. Further studies are required to confirm the direct involvement of the RIPK1/RIPK3/MLKL necroptosis pathway.

**Supplementary Information:**

The online version contains supplementary material available at https://doi.org/10.1007/s12035-026-06178-z.

## Introduction

Carbon tetrachloride (CCL₄) is an industrial liquid that is clear, transparent, heavy, and volatile. CCL₄ has been used as a solvent, dry cleaning fluid, fabric spotting fluid, fire extinguisher fluid, agricultural fumigant, and feedstock for chlorofluorocarbons [1]. Ingestion, cutaneous absorption, or inhalation of its fumes are the three unintentional ways that humans become harmed [2].

CCL₄ is metabolized primarily by the liver, as well as in the kidneys, lungs, and other tissues that contain cytochrome P450 (CYP450) enzymes. CCL₄ can readily penetrate cell membranes due to its lipid solubility. As a result, it accumulates and causes harm not only in the liver but also in the CNS, kidneys, and other organs [3]. The brain is vulnerable to the adverse effects of CCL₄ due to its high levels of polyunsaturated fatty acids (PUFAs), low levels of antioxidant enzymes, and excessive oxygen consumption. However, the precise mechanism of CCL₄-induced neurotoxicity in the brain remains unclear [3, 4].

Necroptosis is a regulated form of programmed cell death mediated mainly by receptor interacting protein kinase 1 (RIPK1), receptor interacting protein kinase 3 (RIPK3), and mixed lineage kinase domain-like protein (MLKL), leading to membrane rupture and inflammation. Necroptosis is a tightly controlled signaling pathway that contributes to inflammatory tissue damage in various pathological conditions [5]. CCL₄ primarily induces apoptosis and necroptosis through oxidative stress and mitochondrial dysfunction via both caspase-3-dependent intrinsic apoptosis and RIPK1-dependent necroptosis [6].

Cerebellar toxicity caused by CCL₄ is a multistep process. Tumor necrosis factor-alpha (TNF-α) causes inflammation by binding to its receptor, TNFR1, establishing Complex I at the membrane [7]. RIPK1 combines with caspase-8 to form Complex IIa, which triggers apoptosis. In the absence of active caspase-8, as can occur under oxidative stress or certain toxic conditions, RIPK1 combines with and activates RIPK3 to create Complex IIb, also known as the necrosome [8]. The sequential development of complex I and IIb leads to necroptosis in TNFα-stimulated cells. Activated RIPK3 recruits and phosphorylates MLKL. Phosphorylated MLKL oligomerizes and migrates to the plasma membrane and affects membrane integrity by generating holes, causing cell enlargement, membrane rupture, and necrotic cell death [9].

Losartan and other angiotensin II receptor antagonists have been proven to control blood pressure in hypertensive patients and to treat diabetic nephropathy [10]. Its advantages in suppressing neuronal damage and enhancing recovery from brain injury point to a favorable safety profile for neurological applications. Numerous models have demonstrated losartan’s neuroprotective properties in epilepsy [11], Alzheimer’s disease [12], and depression [13]. However, until now, losartan has not been evaluated in chemical-induced cerebellar toxicity.

Nanoparticles are particles with sizes between one and one hundred nanometers (nm). Because they are smaller and have a larger surface area relative to their volume, they exhibit different physical and chemical characteristics compared to their bulk counterparts [14, 15].

The goals of this study were to evaluate changes in necroptosis-related marker expression after CCL₄ injection, to assess the potential therapeutic effect of losartan, and to compare the therapeutic efficacy of losartan and losartan potassium nanoparticles (LP-NPs) against CCL₄-induced cerebellar damage.

## Methods

### Calculation of Sample Size

The sample size was determined using G*Power software for Windows (version 3.1.9.2) according to the protocols outlined by Faul et al. [16]. Using previously published quantitative Real time- PCR (qRT-PCR) data for *Ripk3* mRNA expression [17], an effect size (f) of 0.917 was computed. A one-way ANOVA design with a significance level (α) of 0.05 and 90% statistical power yielded a total sample size of 24 rats for the study. As a result, 24 rats were distributed evenly among four experimental groups (*n* = 6 each). The effect size was evaluated using the formula f = σm/σ, where σm is the standard deviation of group means and σ is the within-group standard deviation.

### Animals

The study used twenty-four adult male Sprague–Dawley rats that weighed an average of 200–250 g. They were 9–10 weeks old. Four groups were randomly selected from among the rats: Control (*n* = 6), CCL₄ (*n* = 6), Losartan (*n* = 6), and LP-NPs (*n* = 6)-treated groups. The rats were housed in specially designed metal cages with three rats each, bedding made of soft wood chips, and an atmosphere free of pathogens with controlled humidity levels (60 ± 5%) and temperature ranges of 23ºC ± 3ºC. To acclimate the animals and ensure normal behavior and development, they were fed a commercial basal diet and given unlimited water for two weeks on a 12-h light–dark cycle.

### Ethical Statement

This study was conducted in accordance with the ARRIVE guidelines. All animal experiments were performed in compliance with the National Institutes of Health (NIH) guidelines for the care and use of laboratory animals. The study protocol was approved by the Mansoura University Animal Care and Use Committee (MU-ACUC), Egypt, Approval Code: [MU-ACUC (VM.R.22.11.33)].

### Drugs and Chemicals

CCL₄ (Cat. No. 289116) and losartan potassium (Cat. No. 59489S) were purchased from Sigma-Aldrich Co. (Saint Louis, MO, USA). CCL₄ was diluted 1:1 with olive oil (Egyptian local market) and administered intraperitoneally at 2 mL/kg twice weekly for six weeks [18, 19]. Losartan potassium nanoparticles were synthesized as carrier-free drug nanocrystals (LP-NPs) via top-down mechanical milling without polymers, lipids, or surfactants. Pure losartan potassium was milled in a Retsch MM2000 swing ball mill (China, supplied from the USA) (10 cm^3^ jar, two 12 mm/7 g stainless steel balls) at 20–25 Hz for 30 min (< 30 °C). Particle morphology was verified by transmission electron microscopy [19]. For oral administration, free losartan and LP-NPs were uniformly dispersed in tap water to deliver the therapeutic equivalent dose of 10 mg/kg/day [20, 21].

### Experimental Design

Four groups of six rats each were randomly assigned. The control group received intraperitoneal injections of olive oil (1 mL/kg) twice weekly for 6 weeks. Four weeks after the final dose, the animals were euthanized. The CCL₄ group received intraperitoneal injections of CCL₄ (2 mL/kg, diluted 1:1 in olive oil) twice weekly for 6 weeks, followed by a 4-week observation period before euthanasia. The Losartan-treated group received CCL₄ as described above for 6 weeks, followed by oral administration of losartan potassium at a dose of 10 mg/kg/day for 4 weeks. The LP-NPs-treated group received CCL₄ in the same manner for 6 weeks, followed by oral administration of LP-NPs at a dose of 10 mg/kg/day for 4 weeks, after which all animals were euthanized.

This study utilized a post-injury therapeutic design. After six weeks of CCL₄-induced cerebellar damage, rats were given oral losartan potassium or losartan-loaded nanoparticles for an additional four weeks. To verify that the experimental circumstances and duration were equal, the control and CCL₄ groups were given a four-week maintenance period without treatment. All rats were euthanized at the end of the 10-week experiment.

### Tissue Collection

To achieve total sedation before sacrifice, rats received intraperitoneal injections of 15 mg/kg xylazine and 90 mg/kg ketamine, followed by decapitation. The brain was carefully taken out of the skull and allowed to cool on ice. Both the medulla and pons were elevated. The pons was then removed using forceps. The colliculus inferior and cerebellum were meticulously isolated. The brain was divided into two hemispheres: one was for histopathological analysis in which tissues were fixed in 10% neutral-buffered formalin. The other hemisphere was used for oxidative stress and gene expression analysis. For oxidative stress biomarkers, weighed aliquots (~ 50–100 mg) of tissue were homogenized in ice-cold phosphate-buffered saline (PBS, pH 7.4) at a ratio of 1:10 (w/v) using a glass-Teflon or motorized homogenizer. Homogenization was performed in an ice bath to preserve biomarker integrity. Homogenates were centrifuged at 10,000–20,000 × g for 15–30 min at 4 °C. Supernatants were collected for biochemical assays of oxidative stress biomarkers. For gene expression, an adjacent tissue aliquot (50 mg) is preserved in 500 µL of RNAlater (Qiagen, Germany) and stored at –80 °C until further analysis.

### Biochemical Studies

#### Oxidative Stress Biomarkers

Cerebellar tissue homogenates were analyzed to assess oxidative stress parameters. Lipid peroxidation was determined by measuring malonodialdehyde (MDA) using the thiobarbituric acid-reactive substances (TBARS) method [22]. Reduced glutathione (GSH) levels were quantified according to Beutler’s method [23]. Catalase (CAT) activity was evaluated following the procedure of Fakunle et al. [24], while superoxide dismutase (SOD) activity was assessed using the method described by Struzynska and Toczylowska [25]. All assays were performed using commercially available kits (BioDiagnostic, Egypt).

#### Quantitative Real-time PCR for Assessing mRNA Expressions of *Tnf-α*, *Ripk1*, *Ripk3*, and *Mlkl*

Quantitative real-time PCR (qRT-PCR) was performed to evaluate the mRNA expression of *Tnf-α, Ripk*1*, Ripk*3*,* and *Mlkl*. Total RNA was extracted using QIAzol reagent (Qiagen, Germany) following the manufacturer’s instructions [26]. The concentration, purity, and overall quality of the isolated RNA were assessed by measuring the absorbance ratios (A260/A280 and A260/A230) using a NanoDrop spectrophotometer (Thermo Fisher Scientific, USA). Only samples with acceptable purity ratios were used for downstream analysis.

For cDNA synthesis, 1 µg of purified RNA from each sample was reverse transcribed using the SensiFAST cDNA Synthesis Kit (Bioline, UK). The resulting cDNA was stored at − 20 °C until amplification. Quantitative PCR amplification was carried out on an Applied Biosystems 7500 Real-Time PCR System (Applied Biosystems 7500, USA). Each reaction was performed in a total volume of 20 µL, consisting of 10 µL HERA SYBR Green PCR Master Mix (Willowfort, UK), 2 µL cDNA template, 1 µL each of forward and reverse primers (10 pmol/µL), and 6 µL nuclease-free water. Primers were designed using the Primer3 software (v.4.1.0; http://primer3.ut.ee). All primers were verified for specificity using NCBI BLAST (Primer-BLAST) (https://www.ncbi.nlm.nih.gov/tools/primer-blast/primertool). Thermal cycling conditions included an initial denaturation at 98 °C for 2 min, followed by 45 cycles of denaturation at 95 °C for 10 s and annealing/extension at 60 °C for 30 s. A melt curve analysis was performed at the end of each run to confirm the specificity of amplification and to exclude primer-dimer formation. Each sample was analyzed in technical duplicate, and the mean Ct value was used for downstream analysis. No-template controls (NTC) were included in each qPCR run to monitor contamination and non-specific amplification. Representative amplification plots and melt curve analyses for all primer pairs are provided in Supplementary Figure S1.

The genes of interest (*Tnf-α*, *Ripk*1, *Ripk*3, and *Mlkl*) and the endogenous reference gene (*Gapdh*) are listed with their primer sequences and amplicon lengths in Table 1. Cycle threshold (Ct) values were automatically generated by the instrument’s software using identical threshold settings across all samples. Relative Quantification (RQ) of the gene expression was calculated using the (2^−ΔΔCT^) method [27] after confirming the stable expression of *Gapdh* across all experimental groups.

**Table 1:** The rat primer sequences utilized in qRT-PCR analysis

Gene	Primers	Full gene name	Product length	Reference sequence
*Tnf-α*	F: 5'-TCTTCAAGGGACAAGGCTGC-3' R:5'-CTTGATGGCAGAGAGGAGGC-3'	Tumor necrosis factor alpha	104 bp	NM_012675.3
*Ripk1*	F: 5'-GCAGACCAACCCAGAAGACA-3' R: 5'-CTTGGAGGTAAGGGCACACA-3'	Receptor interacting serine/threonine kinase 1	189bp	NM_001107350.1
*Ripk3*	F: 5'-TAGTTTATGAAATGCTGGACCGC-3' R: 5'-GCCAAGGTGTCAGATGATGTCC-3'	Receptor interacting serine/threonine kinase 3	145 bp	NM_139342.1
*Mlkl*	F: 5'-CCTGGAACTCGGGGTATGGA-3' R:5'-GCACACGGTTTCTTAGACGC-3'	Mixed lineage kinase domain like pseudokinase	121bp	NM_001401077.1
*Gapdh*	F:5'-CCTCGTCTCATAGACAAGATGGT-3' R: 5'-GGGTAGAGTCATACTGGAACATG-3'	Glyceraldehyde-3-phosphate dehydrogenase	169 bp	NM_017008.4

### Histological and Immunohistochemical Stains

Sections of the cerebellum, each 5 µm thick, were separated. The laterally extended sections were cut after the medial sagittal section. The slices were labeled and stained with hematoxylin and eosin (H&E), Luxol Fast Blue (LFB) [28], and immunohistochemically. Before immunohistochemical labeling, the cerebellar slides were treated with 3% hydrogen peroxide to suppress endogenous peroxidase activity. Following three PBS (pH 7.4) rinses, the sections were treated in sodium citrate buffer (0.01 M, pH 6.0) in a water bath at 95 °C for 30 min to recover antigens. 1% bovine serum albumin (BSA) was applied to the sections for an hour after they had been allowed to come to room temperature. Next, the primary antibodies were added (4 °C, overnight): anti-RIPK1 (ab72139, Abcam, Cambridge, UK, RRID:AB_2194363, 1:200), anti-caspase-8 (ab25901, Abcam, Cambridge, UK, RRID:AB_2287097, 1:200), anti-RIPK3 (ABIN2792102, RRID:AB_2925703, 1:200), anti- Glial Fibrillary Acidic Protein (GFAP) (ab7260, Abcam, Cambridge, UK; RRID:AB_305808, 1:1000), and anti- Ionized calcium-binding adaptor molecule (Iba-1) (ab5076, Abcam, Cambridge, UK; RRID:AB_2224402, 1:1000) [17, 29, 30]. Then, anti-Ms/Rb polymer-HRP (Biocyc Biotechnologie, Germany, DSK-211–50), a ready-to-use secondary antibody, was added. The following day, the slides were cleaned in phosphate-buffered saline and then incubated. Brown immunoreactivity was observed following the application of diaminobenzidine (DAB) staining to the slides using the mouse and rabbit HRP/DAB (ABC) detection IHC kit (ab64264, Abcam, UK). To counterstain the sections, hematoxylin was used. All histological scoring, immunohistochemical quantification, morphometric measurements, and image analyses were performed by investigators blinded to the treatment groups to minimize observer bias.

### Morphometric Study

Morphometric analysis was performed using ImageJ and Fiji software [31]. Six rats per group were included for each immunohistochemical marker (RIPK1, caspase-8, RIPK3, GFAP, and Iba1). For each animal, two cerebellar sections were analyzed, and two non-overlapping fields (× 400) were randomly selected per section. Accordingly, four fields per animal and a total of 24 fields per group were evaluated. For each animal, measurements from four microscopic fields (two sections per animal and two fields per section) were averaged to obtain a single representative value. These animal-based mean values (*n* = 6 per group) were used for statistical analysis. The percentage area of immunopositive staining was calculated for each field and averaged for statistical analysis. The photos were then put through the following tests:

- The normal Purkinje cell number per field was determined by analyzing H&E sections (magnification: × 200).
- The myelination of the Luxol Fast Blue (LFB)-stained sections was quantitatively evaluated using the area percentage (magnification: × 400). A black binary color was used to mask and quantify the LFB-stained region.
- Assessment of the immunohistochemistry expression of RIPK1, RIPK3, Caspase-8, GFAP, and Iba1. Positive responses were associated with the percentage of brown pixels that filled the area in the high-power fields (magnification: × 400). By selecting "H DAB" from the plugin list and utilizing color deconvolution, three new images were generated. Hematoxylin was represented by color 1, DAB by color 2, and a complementary picture, color 3, which included colors of both DAB and hematoxylin. For quantification, the DAB pictures were employed. An area fraction based on predetermined measurements was selected from the analysis list [32, 33].

### Statistical Analysis

Data entry and analysis were performed using IBM SPSS Statistics for Windows, version 26.0 (IBM Corp., Armonk, NY, USA, 2019). The Shapiro–Wilk test was applied to assess normality, with a *P* value > 0.05 indicating normally distributed data. Outliers were identified using boxplots. Quantitative variables were expressed as mean ± standard deviation (SD). Group comparisons were conducted using one-way analysis of variance (one-way ANOVA) for parametric data, followed by Tukey’s post hoc test for multiple comparisons with a 95% confidence interval. Statistical significance was set at *P* ≤ 0.05.

## Results

### Assessment of Oxidative Stress Biomarkers

**Fig. 1:**
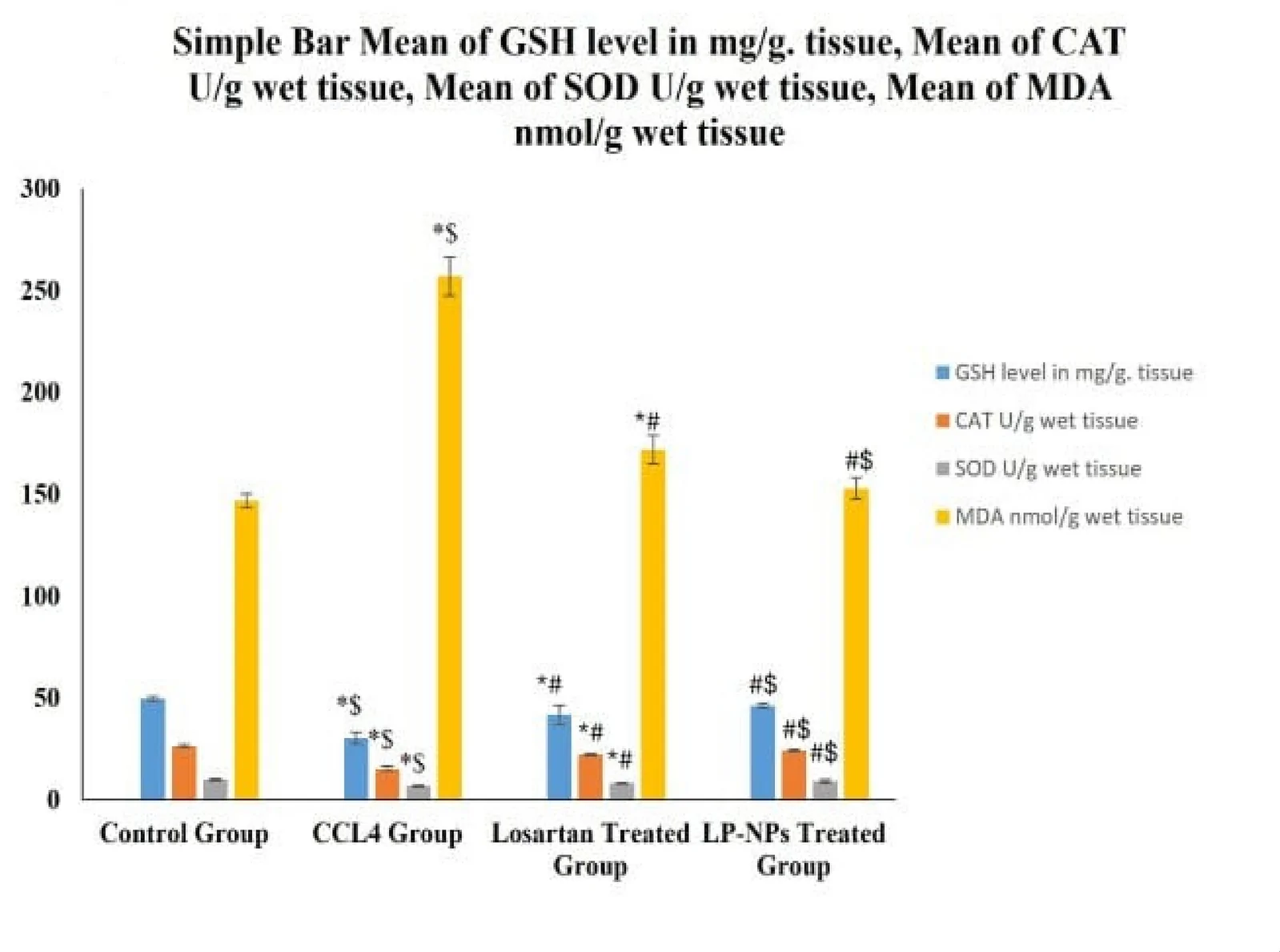
Levels of GSH, CAT, SOD, and MDA in cerebellar tissue homogenates across the experimental groups. Data represent mean ± SD (*n* = 6 animal/group); Statistical analysis was performed by one-way ANOVA with Tukey’s post hoc test. *P ≤ 0.05 vs. control; #*P* ≤ 0.05 vs. CCL_4_; $P ≤ 0.05 vs. Losartan

Cerebellar GSH, CAT, and SOD levels were significantly lower in the CCL₄ group than in the control group (*P* ≤ 0.05). Compared to the CCL₄ group, the Losartan and LP-NPs-treated groups had significantly higher levels of GSH, CAT, and SOD (*P* ≤ 0.05). GSH, CAT, and SOD levels significantly improved between the Losartan and LP-NPs-treated groups (*P* ≤ 0.05). The CCL₄ group had significantly higher cerebellar MDA levels than the control group (*P* ≤ 0.05). When compared to the CCL₄ group, MDA levels in the Losartan and LP-NPs- treated groups dropped significantly (*P* ≤ 0.05). Furthermore, MDA levels were significantly lower (*P* ≤ 0.05) in rats treated with the LP-NPs than in those treated with losartan (Fig. 1).

### Quantitative Real Time- PCR (qRT-PCR) for *Tnf-α*, *Ripk3*, *Ripk1*, and *Mlkl* mRNA Expressions

To validate the use of *Gapdh* as an endogenous control, its Ct values were compared across all experimental groups. *Gapdh* expression showed no statistically significant variation among the study groups (*P* > 0.05), indicating stable transcription under all treatment conditions. Therefore, *Gapdh* was considered an appropriate reference gene for normalization of target gene expression in the present study.

**Fig. 2:**
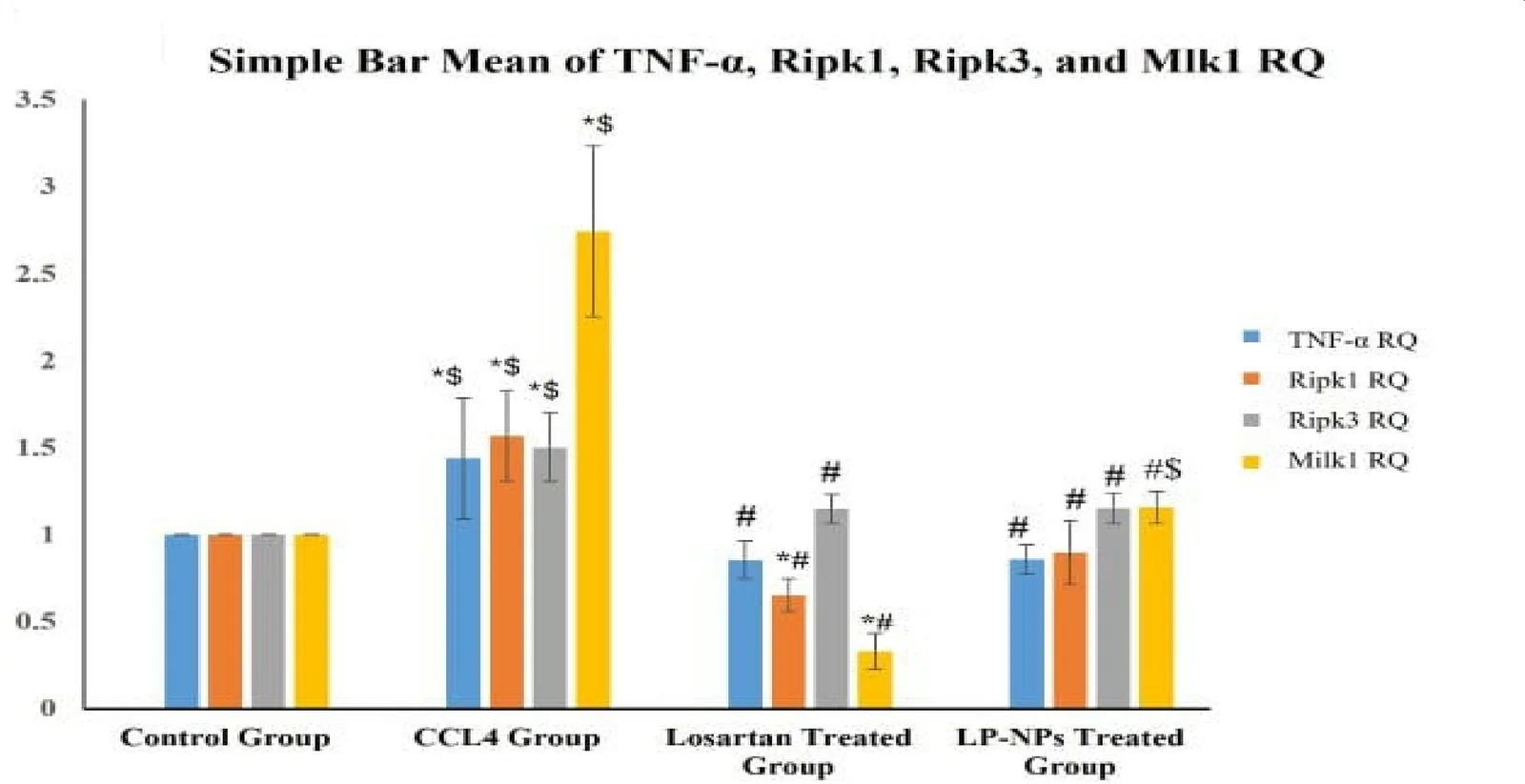
Relative mRNA expression levels of *Tnf-α, Ripk1, Ripk3*, and *Mlkl* in cerebellar tissue across the experimental groups. Data represent mean ± SD (*n* = 6 animal/group); Statistical analysis was performed by one-way ANOVA with Tukey’s post hoc test. *P ≤ 0.05 vs. control; #*P* ≤ 0.05 vs. CCL_4_; $P ≤ 0.05 vs. Losartan

The expressions of *Tnf-α, Ripk1, Ripk3*, and *Mlkl* mRNA were considerably greater in the CCL₄ group (*Tnf-α*: 1.44 ± 0.33, *Ripk1*: 1.57 ± 0.25, *Ripk3*: 1.503 ± 0.188, and *Mlkl:* 2.74 ± 0.47) compared to the control group (*P* ≤ 0.05). Treatment with either conventional losartan or LP-NPs significantly reduced the mRNA expression of *Tnf-α* (0.855 ± 0.103 and 0.859 ± 0.079, respectively), *Ripk1* (0.652 ± 0.091 and 0.896 ± 0.173, respectively), *Ripk3* (1.149 ± 0.081 and 1.152 ± 0.082, respectively), and *Mlkl* (0.329 ± 0.098 and 1.157 ± 0.086, respectively) compared to the CCL₄ group (*P* ≤ 0.05). No statistically significant differences were observed between the Losartan- and LP-NPs-treated groups for *Tnf-α, Ripk1*, or *Ripk3* gene expressions (*P* > 0.05). In contrast, *Mlkl* mRNA expression was significantly lower in the Losartan-treated group than in the LP-NPs-treated group (*P* ≤ 0.05) (Fig. 2).

### Histopathological Assessment

#### Hematoxylin and Eosin-stained Sections

**Fig. 3:**
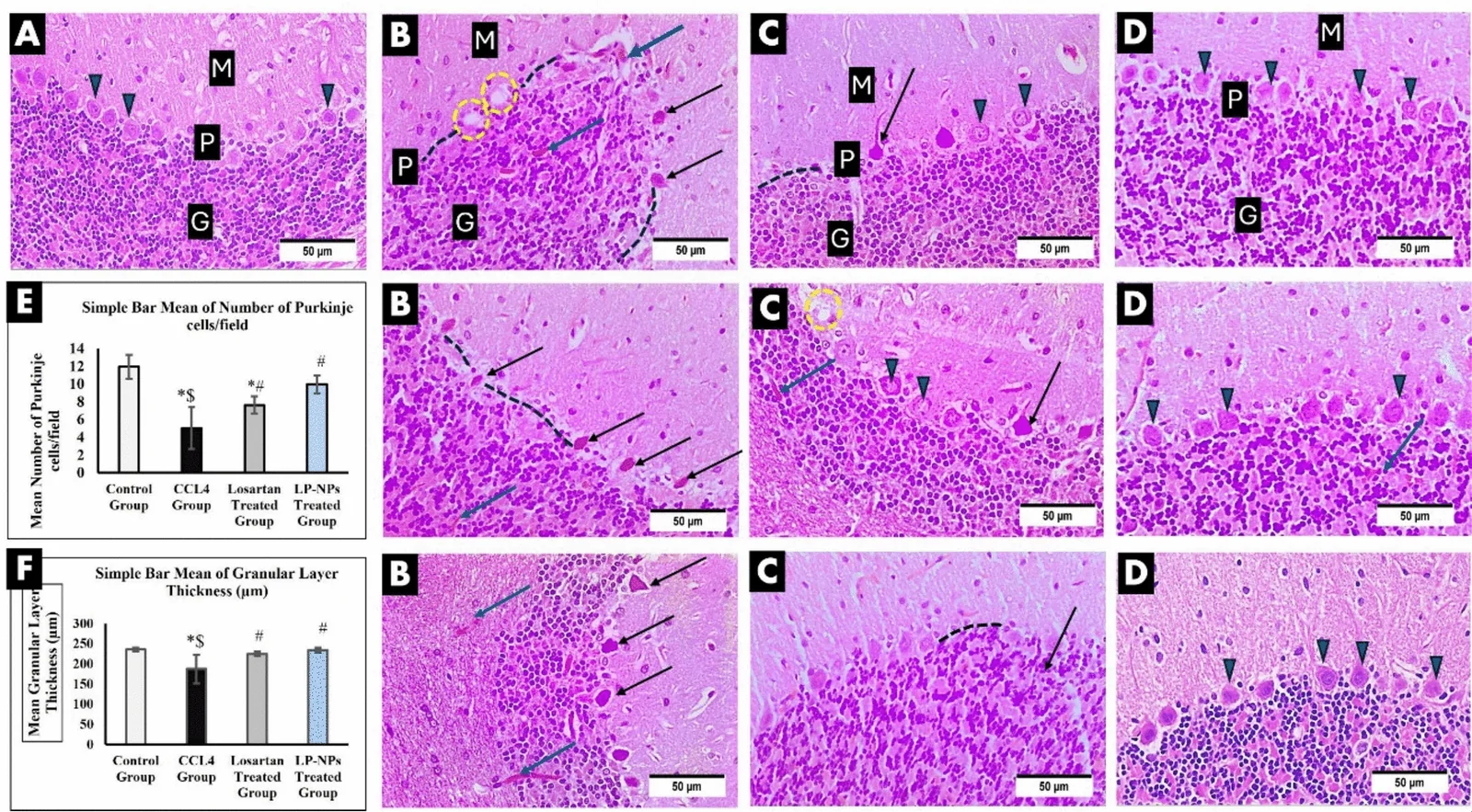
Representative H&E-stained cerebellar sections (× 400) and morphometric analyses. **A** The Control group showing intact molecular (M), Purkinje cell (P), and granular (G) layers with normal Purkinje cells (arrowheads). **B** The CCL₄ group showing cortical disruption, shrunken Purkinje cells (black arrows), vacuolation (yellow dotted circles), Purkinje layer discontinuity (black dotted lines), and hemorrhage (blue arrows). **C** The Losartan-treated group showing partial cortical restoration, Purkinje cells with vesicular nuclei (arrowheads) or eosinophilic shrinkage, and reduced layer discontinuity and hemorrhage. **D** The LP-NPs-treated group showing continuous Purkinje cell layer (arrowheads) without overt hemorrhage. **E**, **F** Quantitative Purkinje cell counts and granular layer thickness (µm), respectively. Data represent mean ± SD (*n* = 6 animal/group); Statistical analysis was performed by one-way ANOVA with Tukey’s post hoc test. *P ≤ 0.05 vs. control; #*P* ≤ 0.05 vs. CCL_4_; $P ≤ 0.05 vs. Losartan

Histological examination of H&E-stained cerebellar sections from the control group demonstrated preserved anatomical organization across the three cortical layers: the internal granular, Purkinje, and external molecular layers. The granular layer contained densely packed granule cells, while the Purkinje layer was arranged as a single continuous monolayer of pyriform cells featuring large cell bodies, pale round nuclei, and prominent nucleoli. The molecular layer contained scattered cellular elements and neuronal processes (Fig. 3A).

In contrast, the CCL_4_ group exhibited disruption of cortical architecture. The continuous monolayer arrangement of Purkinje cells was altered, with focal regions displaying cellular loss and disorganization. Degenerative cellular changes were evident, characterized by cytoplasmic shrinkage and nuclear pyknosis with intense eosinophilia (Fig. 3B). Sections from the Losartan-treated group showed partial attenuation of tissue alterations, though focal cytoplasmic vacuolation, microscopic hemorrhagic foci, and localized Purkinje cell loss remained observable (Fig. 3C). In the LP-NPs-treated group, Purkinje cells maintained monolayer continuity and pyriform cellular structure across examined fields (Fig. 3D).

Quantitative analysis (× 200) demonstrated a significant reduction in Purkinje cell counts in the CCL₄ group (5.033 ± 2.253 cells/field) compared to the control group (11.933 ± 1.275 cells/field, *P* ≤ 0.05). The Losartan-treated group (7.633 ± 0.915 cells/field) also showed lower counts than the control group (*P* ≤ 0.05) but higher than the CCL₄ group. The LP-NPs-treated group (9.967 ± 0.958 cells/field) showed significantly higher counts than the CCL₄ group (*P* ≤ 0.05) and did not differ significantly from the Losartan-treated group (*P* > 0.05) (Fig. 3E).

Similarly, granular layer thickness was significantly reduced in the CCL₄ group (187.777 ± 33.947 µm) compared to the control group (235.805 ± 4.426 µm, *P* ≤ 0.05). The Losartan-treated group (225.030 ± 4.965 µm) showed significantly greater thickness than the CCL₄ group (*P* ≤ 0.05), though not significantly different from the control group (*P* > 0.05). Treatment with LP-NPs restored thickness (234.825 ± 5.385 µm), with no difference from the control group (*P* > 0.05) but significantly higher values than the CCL₄ group (*P* ≤ 0.05). (Fig. 3F).

#### Luxol Fast Blue (LFB)-stained Sections

**Fig. 4:**
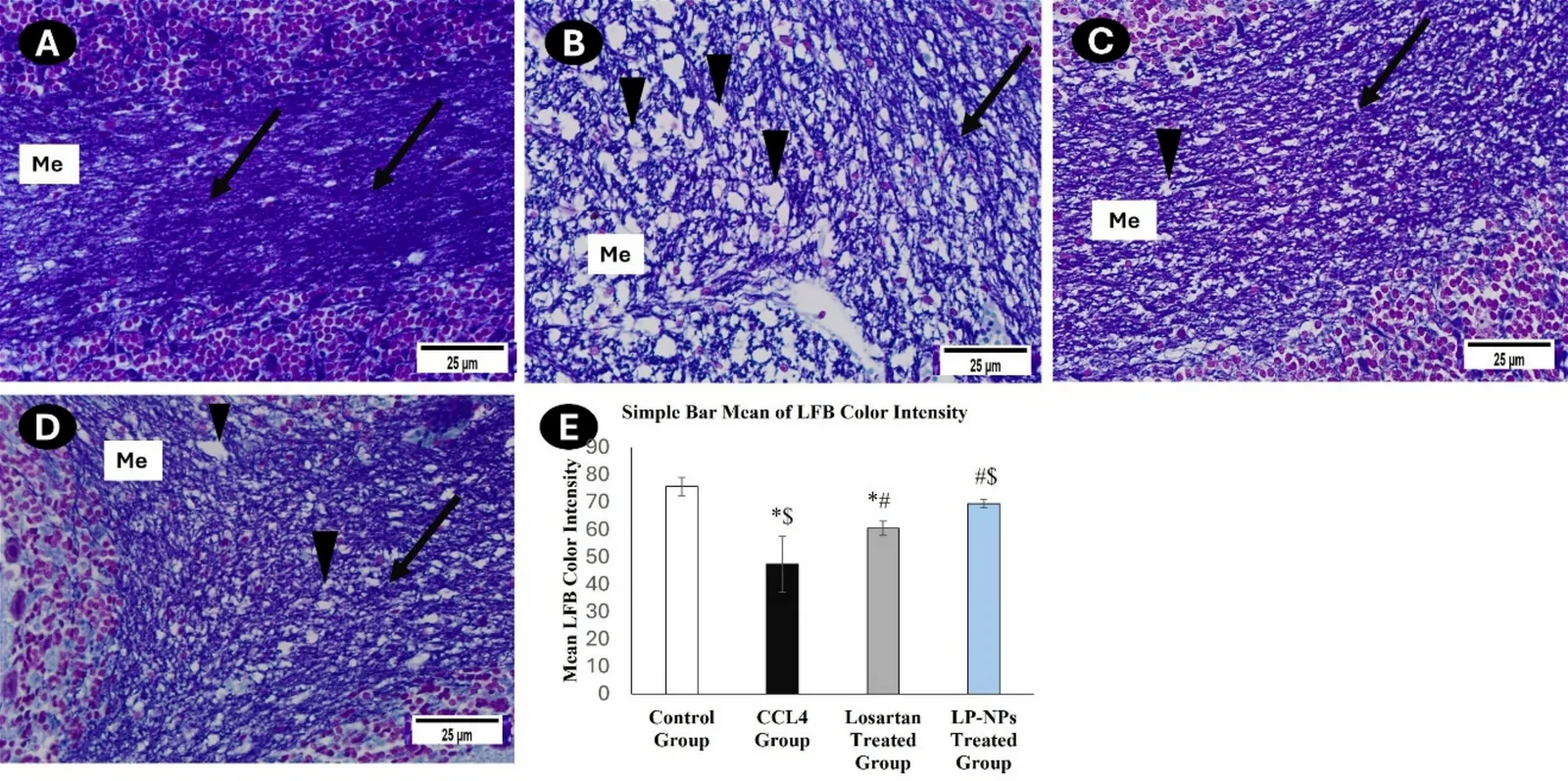
Representative Luxol Fast Blue (LFB)-stained cerebellar sections (× 400) and morphometric analysis. **A** The Control group showing thick, dark blue myelinated nerve fiber bundles (arrows) in the cerebellar medulla (Me). **B** The CCL₄ group showing myelin disruption, irregular vacuolation (arrowheads), and loosely arranged, lightly stained nerve fibers (arrows). **C** The Losartan-treated group showing reduced vacuole size (arrowheads) and increased density of myelinated fibers. **D** The LP-NPs-treated group showing densely packed, intensely blue-stained fiber bundles (arrows) with minimal vacuolation (arrowheads). **E** Quantification of LFB staining intensity in white matter. Data represent mean ± SD (*n* = 6 animal/group); Statistical analysis was performed by one-way ANOVA with Tukey’s post hoc test. *P ≤ 0.05 vs. control; #*P* ≤ 0.05 vs. CCL_4_; $P ≤ 0.05 vs. Losartan

In control sections stained with Luxol Fast Blue (LFB), dark blue-stained, continuous myelinated nerve fiber bundles were observed within the cerebellar white matter core (Fig. 4A). In the CCL_4_ group, white matter tracts exhibited pallor and structural disruption of myelinated fibers, accompanied by irregular, variably sized parenchymal vacuolation (Fig. 4B). The Losartan-treated group showed reduced vacuolation size and increased density of blue-stained fibers relative to the CCL_4_ group (Fig. 4C). The LP-NPs-treated group displayed densely packed, blue-stained nerve fiber bundles with minimal vacuolation across cerebellar tracts (Fig. 4D).

Morphometric analysis revealed that the color intensity of LFB-stained nerve fibers in the CCL₄ group was significantly lower (54.391 ± 9.786) than in the control group (75.688 ± 3.234) (*P* ≤ 0.05). The groups treated with conventional losartan (60.505 ± 2.506) and LP-NPs (69.478 ± 1.402) demonstrated a significant increase (*P* ≤ 0.05) in comparison to the CCL₄ group. Additionally, it was found that the LP-NPs-treated group showed more LFB staining intensity in cerebellar white matter than the Losartan-treated group (*P* ≤ 0.05) (Fig. 4E).

#### Immunohistochemical Assessment

##### **RIPK1 Immunoreactivity**

**Fig. 5:**
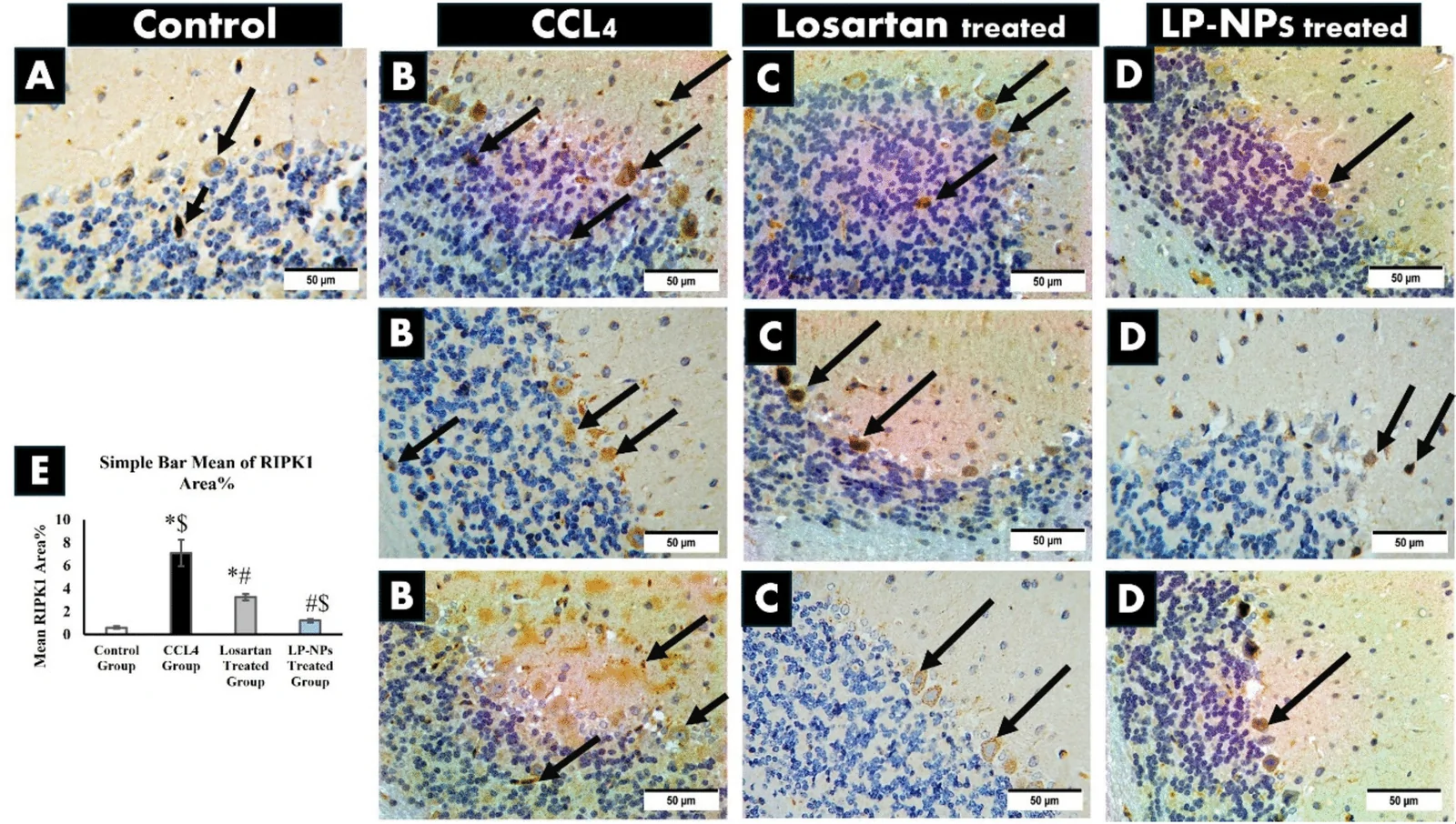
Representative RIPK1 immunohistochemistry (× 400) and quantitative morphometry. **A** The Control group showing weak, sporadic RIPK1 immunopositivity (black arrow). **B** CCL_4_ group showing increased RIPK1 immunoreactivity across cortical layers (black arrows). **C** The Losartan-treated group showing moderate RIPK1 immunopositivity (black arrows). **D** The LP-NPs-treated group showing reduced, weak RIPK1 immunoreactivity (black arrow). **E** Quantification of RIPK1-positive area percentage (%). Data represent mean ± SD (*n* = 6 animal/group); Statistical analysis was performed by one-way ANOVA with Tukey’s post hoc test. *P ≤ 0.05 vs. control; #*P* ≤ 0.05 vs. CCL_4_; $P ≤ 0.05 vs. Losartan

In the control group, RIPK1 immunohistochemistry revealed weak scattered cytoplasmic immunoreactivity across the cortical layers (Fig. 5A). In contrast, the CCL₄ group exhibited prominent RIPK1 immunoreactivity, particularly within the Purkinje cell layer (Fig. 5B). The Losartan-treated group showed moderate immunoreactivity (Fig. 5C), whereas the LP-NPs-treated group displayed localized, weak immunopositivity restricted to isolated Purkinje cells (Fig. 5D).

The average percentage of RIPK1-positive immunoreactions in each field was determined (magnification: × 400). Quantitative analysis showed that the RIPK1-positive area percentage was significantly higher in the CCL₄ group (7.085 ± 1.078%) compared to the control group (0.586 ± 0.0936%, *P* ≤ 0.05). Both Losartan- (3.243 ± 0.264%) and LP-NPs-treated groups (1.207 ± 0.131%) exhibited a significant reduction in the RIPK1-positive area percentage compared to the CCL₄ group (*P* ≤ 0.05). The positive area percentage was significantly lower in the LP-NPs-treated group than in the Losartan-treated group and did not differ significantly from the control group (Fig. 5E).

##### **RIPK3 Immunoreactivity**

**Fig. 6:**
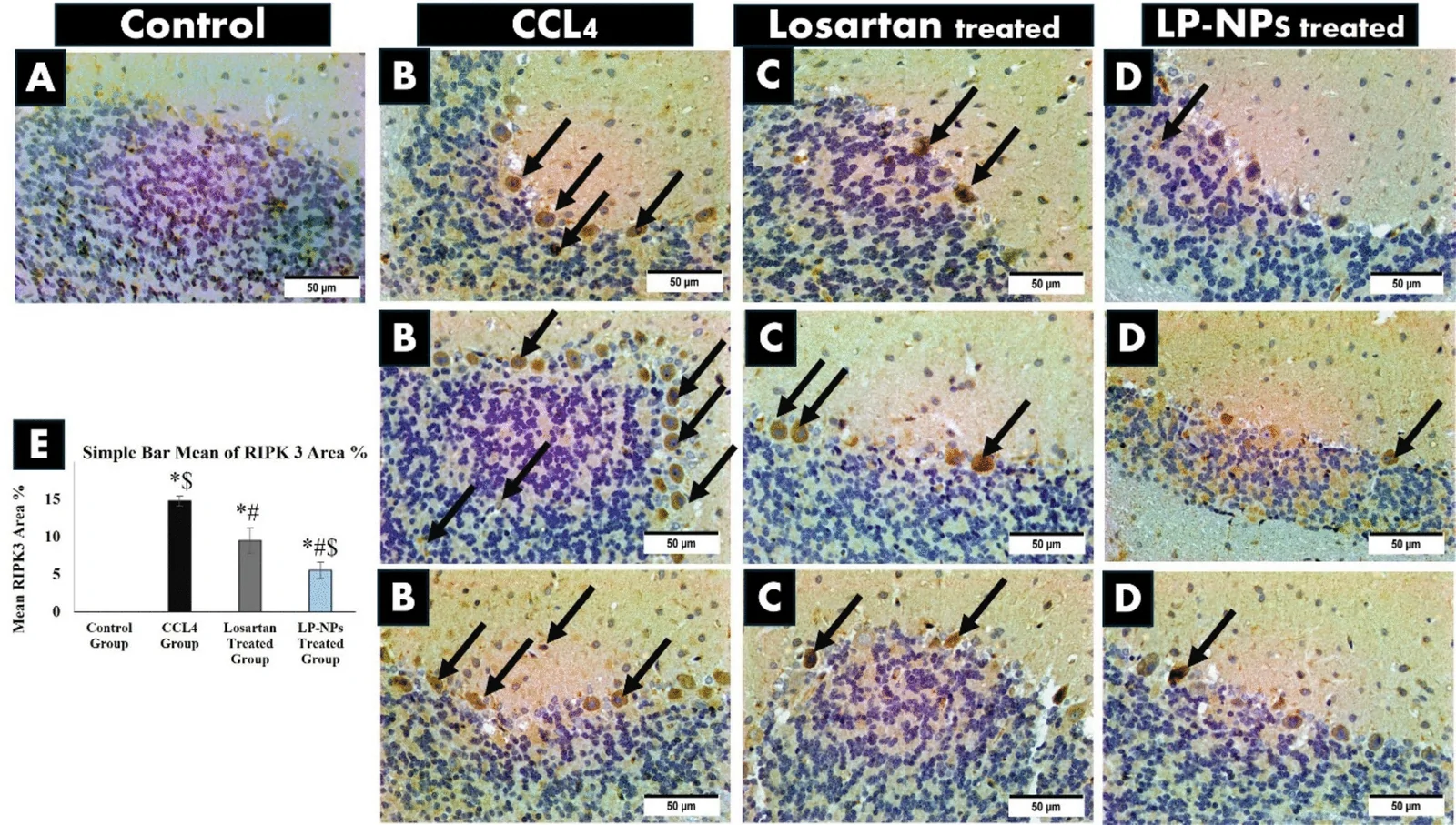
Representative RIPK3 immunohistochemistry (× 400) and quantitative morphometry. **A** The Control group showing weak RIPK3 immunoreactivity (black arrow). **B** The CCL_4_ group showing increased, diffuse RIPK3 immunopositivity (black arrows). **C** The Losartan-treated group showing moderate RIPK3 immunoreactivity (black arrows). **D** The LP-NPs-treated group showing reduced, mild RIPK3 immunopositivity. **E** Quantification of RIPK3-positive area percentage (%). Data represent mean ± SD (*n* = 6 animal/group); Statistical analysis was performed by one-way ANOVA with Tukey’s post hoc test. *P ≤ 0.05 vs. control; #*P* ≤ 0.05 vs. CCL_4_; $P ≤ 0.05 vs. Losartan

RIPK3 immunohistochemistry showed no detectable immunoreactivity in the control group (Fig. 6A). The CCL₄ group demonstrated strong RIPK3 positivity in all layers, particularly in the Purkinje cell cytoplasm (Fig. 6B). Treatment with conventional losartan resulted in moderate positivity (Fig. 6C), while treatment with LP-NPs produced only mild immunoreactivity in some Purkinje cells (Fig. 6D).

Quantification (× 400) revealed that the RIPK3-positive area percentage was significantly higher in the CCL₄ group (14.755 ± 0.649%) compared to the control group (0.0 ± 0.0, *P* ≤ 0.05). Both Losartan (9.498 ± 1.64%) and LP-NPs-treated groups (5.487 ± 1.04%) exhibited a significant lower positive area percentage versus the CCL₄ group (14.755 ± 0.649%) (*P* ≤ 0.05). The LP-NPs-treated group showed a significantly lower positive area percentage than the Losartan-treated group, but both remained higher than the control group (*P* ≤ 0.05) (Fig. 6E).

##### **Caspase-8 Immunoreactivity**

**Fig. 7:**
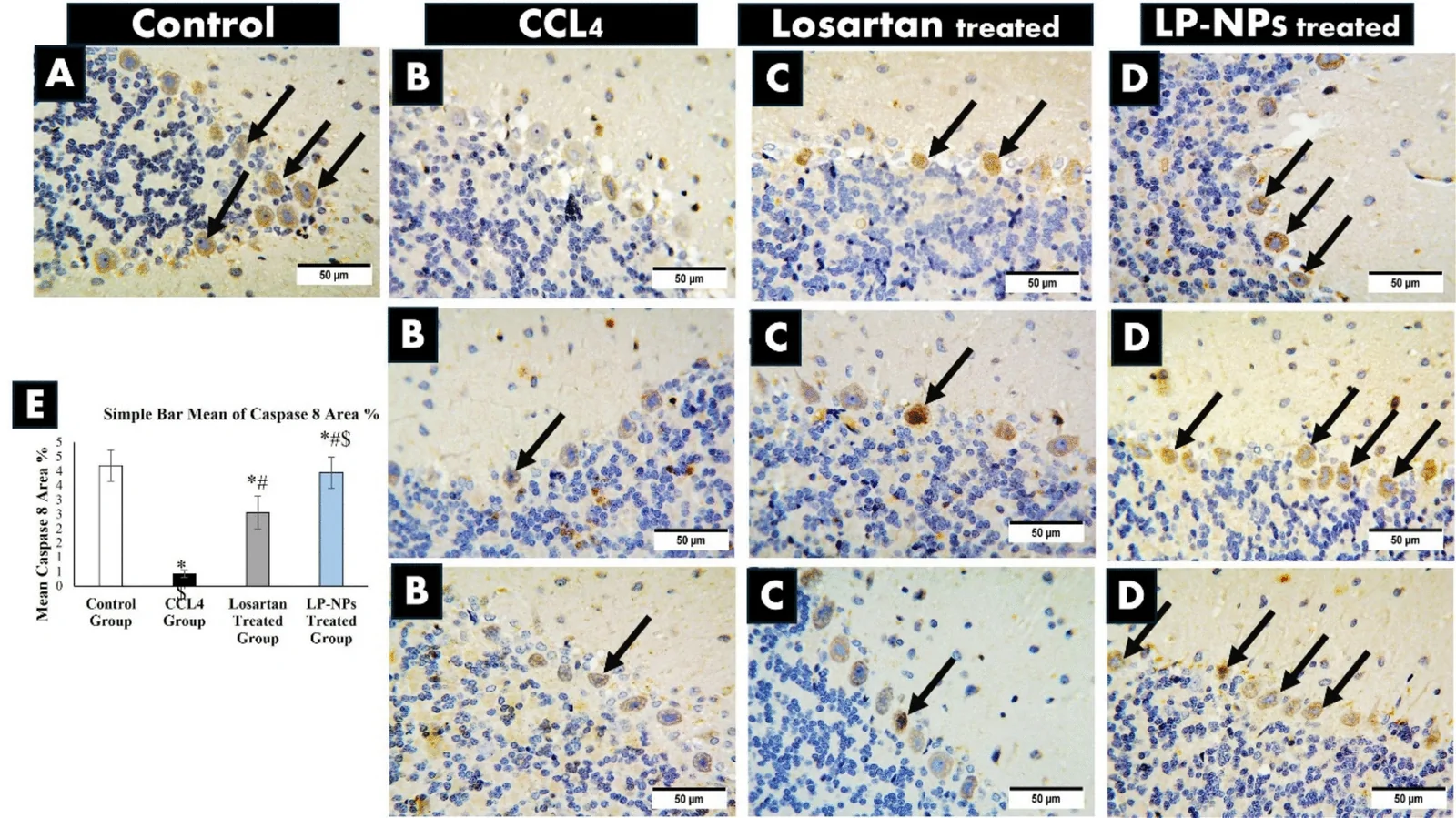
Representative Caspase-8 immunohistochemistry (× 400) and quantitative morphometry. **A** The Control group showing strong cytoplasmic Caspase-8 immunoreactivity in Purkinje cells (black arrows). **B** The CCL_4_ group showing minimal Caspase-8 immunopositivity (black arrows). **C** The Losartan-treated group showing mild Caspase-8 immunoreactivity. **D** The LP-NPs-treated group showing increased Caspase-8 immunoreactivity (black arrows). **E** Quantification of Caspase-8-positive area percentage (%). Data represent mean ± SD (*n* = 6 animal/group); Statistical analysis was performed by one-way ANOVA with Tukey’s post hoc test. *P ≤ 0.05 vs. control; #*P* ≤ 0.05 vs. CCL_4_; $P ≤ 0.05 vs. Losartan

In the control group, caspase-8 immunoreactivity demonstrated strong cytoplasmic positivity (Fig. 7A). The CCL₄ group exhibited a marked reduction, with only very weak immunoreactivity (Fig. 7B). Treatment with conventional losartan partially restored immunoreactivity with mild positivity (Fig. 7C), while treatment with LP-NPs showed a moderately positive area percentage (Fig. 7D).

Quantitative assessment (× 400) showed a significant reduction in caspase-8 positivity in the CCL₄ group (0.423 ± 0.123%) compared to the control group (7.186 ± 0.518%, *P* ≤ 0.05). Both conventional losartan (2.553 ± 0.553%) and LP-NPs (3.943 ± 0.523%) significantly increased positivity compared to the CCL₄ group (*P* ≤ 0.05). LP-NPs induced a significantly higher response than conventional losartan, though both remained lower than the control group (*P* ≤ 0.05) (Fig. 7E).

##### **GFAP Immunoreactivity**

**Fig. 8:**
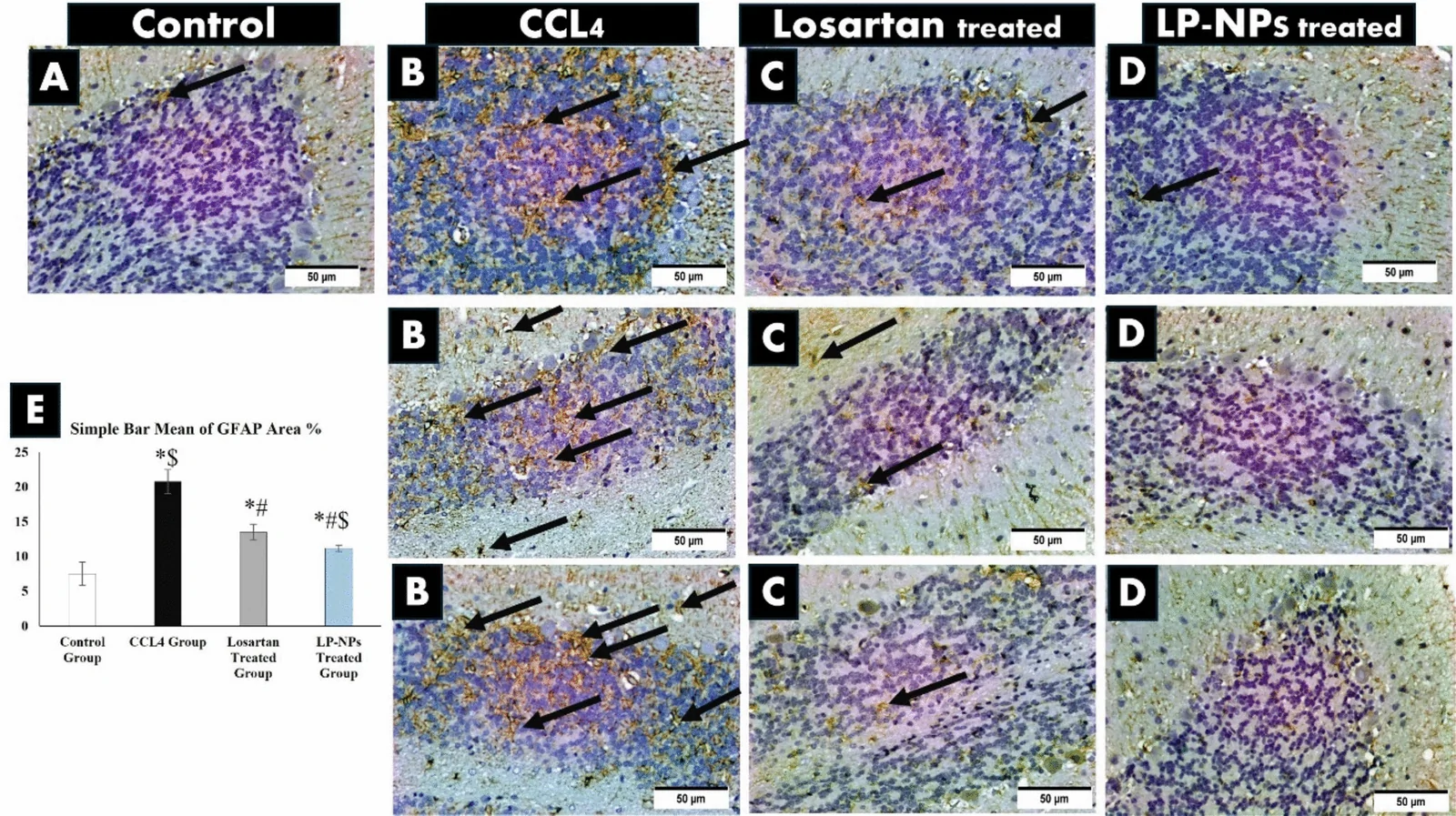
Representative GFAP immunohistochemistry (× 400) and quantitative morphometry. **A** The Control group showing minimal GFAP immunoreactivity in astrocytic processes. **B** The CCL_4_ group showing marked, hypertrophic GFAP immunopositivity (black arrows). **C** The Losartan-treated group showing mild GFAP immunoreactivity. **D** The LP-NPs-treated group showing sparse, weak GFAP immunopositivity (black arrow). **E** Quantification of GFAP-positive area percentage (%). Data represent mean ± SD (*n* = 6 animal/group); Statistical analysis was performed by one-way ANOVA with Tukey’s post hoc test. *P ≤ 0.05 vs. control; #*P* ≤ 0.05 vs. CCL_4_; $P ≤ 0.05 vs. Losartan

Control cerebellar sections revealed thin, delicate GFAP-positive astrocytic processes, with predominant localization in the granular layer (Fig. 8A). The CCL_4_ group exhibited dense, hypertrophic GFAP-positive astrocytic processes across all cortical layers (Fig. 8B). Conventional losartan treatment was associated with reduced astrocytic process density (Fig. 8C), while LP-NPs treatment resulted in sparse, thin GFAP-positive processes (Fig. 8D).

Quantitative analysis (× 400) demonstrated significantly higher GFAP positivity in the CCL_4_ group (20.798 ± 1.62%) compared to the control group (7.499 ± 1.61%, *P* ≤ 0.05). Both conventional losartan (13.517 ± 1.06%) and LP-NPs (11.165 ± 0.444%) significantly reduced positive area percentage compared to the CCL₄ group (*P* ≤ 0.05). LP-NPs-treated group showed a significantly lower positive area percentage than Losartan-treated group (*P* ≤ 0.05) (Fig. 8E).

##### **Iba1 Immunoreactivity**

**Fig. 9:**
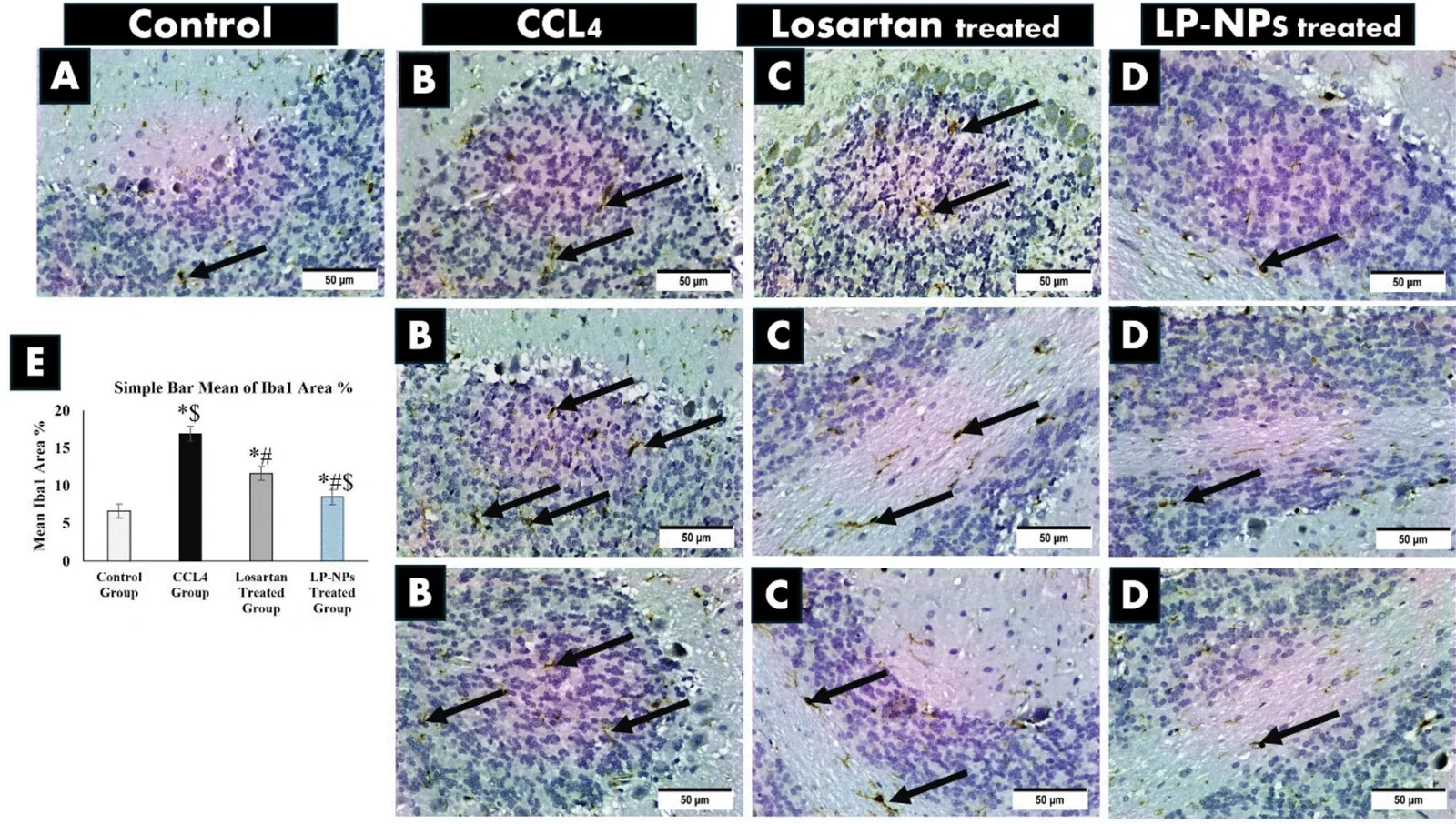
Representative Iba1 immunohistochemistry (× 400) and quantitative morphometry. **A** The Control group showing sparse Iba1-positive microglia. **B** The CCL_4_ group showing marked Iba1 immunoreactivity with altered microglial morphology (black arrows). **C** The Losartan-treated group showing moderate Iba1 immunopositivity (black arrows). **D** The LP-NPs-treated group showing weak, focal Iba1 immunoreactivity (black arrow). **E** Quantification of Iba1-positive area percentage (%). Data represent mean ± SD (*n* = 6 animal/group); Statistical analysis was performed by one-way ANOVA with Tukey’s post hoc test. *P ≤ 0.05 vs. control; #*P* ≤ 0.05 vs. CCL_4_; $P ≤ 0.05 vs. Losartan

In the control group, Iba1 immunostaining showed sparse, ramified microglial cells distributed within the gray matter (Fig. 9A). The CCL_4_ group exhibited dense Iba1 immunopositivity characterized by microglial cell bodies with thickened processes (Fig. 9B). Conventional losartan treatment produced moderate Iba1 positivity (Fig. 9C), whereas LP-NPs treatment was associated with mild, focal Iba1 immunoreactivity (Fig. 9D).

Quantification (× 400) revealed significantly higher Iba1 positivity in the CCL₄ group (16.908 ± 0.932) compared to the control group (6.646 ± 0.876, *P* ≤ 0.05). Both LP-NPs (8.479 ± 0.96) and losartan (11.646 ± 0.875) significantly reduced the Iba1 positive area percentage relative to the CCL₄ group (*P* ≤ 0.05). The LP-NPs-treated group showed a significantly lower response than the Losartan-treated group (*P* ≤ 0.05) (Fig. 9E).

## Discussion

CCL₄ is a hazardous chemical that is used in freezers, fire extinguishers, dry cleaners, and other locations. CCL₄ causes oxidative damage by producing free radicals in several organs, including the kidney, heart, brain, liver, and lungs [34]. The cerebellum, which plays a crucial role in several brain functions, has received considerable attention in neuroscience studies [35]. Losartan’s neuroprotective potential has been demonstrated across various preclinical models of secondary neural injury [12]. Our study was designed to investigate two intriguing research topics: the neurotoxic effects of CCL₄ on the cerebellum and the associated changes in necroptosis-related markers and the potential protective impact of conventional losartan, as well as whether LP-NPs have an additional promoting influence on it.

CCL₄ significantly raised MDA levels compared to the control group, indicating elevated lipid peroxidation. Lipid peroxidation, facilitated by the generation of CCL₄ free radical derivatives, is one of the primary causes of CCL₄-induced toxicity. According to Makni et al. [36], CYP450 transforms CCL₄ into the highly reactive trichloromethyl radical (CCl_3_•). When CCl_3_• reacts rapidly with oxygen, the highly reactive trichloromethyl peroxyl radical (CCl_3_OO•) is created and reacts with lipids to create lipid peroxides; this radical starts a chain reaction. The brain’s high oxygen demand and high polyunsaturated fat content, which oxygen free radicals can break down, render it vulnerable to this type of oxidative stress [37].

It has been proven that peroxidases, catalase, and SOD are enzymes that assist cells in protecting against oxygen cytotoxicity. SOD catalyzes the dismutation process that produces hydrogen peroxide (H_2_O_2_), while catalase or peroxidase removes it [38]. In our study, CCL₄ decreased levels of catalase and SOD. These findings are consistent with those of Alam [39] and Makni et al. [36] in which CCL₄ was associated with excessive free radical generation and impairment of endogenous antioxidant defense mechanisms.

In comparison to the CCL₄ group, the oxidative stress condition improved in the Losartan- and LP-NPs-treated groups, as evidenced by a significant decrease in MDA levels and a significant increase in SOD, GSH, and catalase levels. These findings are consistent with prior research suggesting antioxidant effects for losartan in other modalities, such as cardiotoxicity [40] and aging [41] due to their ability to counteract oxidative stress and restore endogenous antioxidant defense mechanisms. LP-NPs produced greater improvements than conventional losartan in the measured oxidative stress parameters.

The present study evaluated key markers associated with necroptosis signaling. CCL₄ significantly upregulated the mRNA expression of *Tnf-α, Ripk1, Ripk3*, and *Mlkl*. Both conventional losartan and LP-NPs significantly downregulated *Tnf-*α*, Ripk1, Ripk3*, and *Mlkl* mRNA expression relative to the CCL_4_ group. However, molecular gene expression analysis did not demonstrate LP-NPs superiority over conventional losartan. Specifically, no statistically significant differences were detected between the two treated groups regarding *Tnf-*α*, Ripk1, and Ripk3* mRNA levels, whereas conventional losartan produced a greater reduction in *Mlkl* mRNA expression than LP-NPs.

In addition, CCL₄ treatment was accompanied by lower caspase-8 immunoreactivity and higher RIPK1 and RIPK3 immunoreactivity, as observed by semi-quantitative immunohistochemical analysis, all of which were significantly altered following losartan and LP-NPs administration, with LP-NPs showing greater improvement specifically in selected histological and immunohistochemical alterations induced by CCL₄.

However, altered immunoreactivity and mRNA expression of RIPK1, RIPK3, and caspase-8 reflect changes in total marker levels rather than definitive pathway activation or inhibition. Functional activation of necroptosis typically requires demonstration of phosphorylated forms (such as p-RIPK3 and p-MLKL) or pathway-specific inhibition studies. Therefore, these findings demonstrate that CCL₄ toxicity and subsequent treatments are associated with altered expression of necroptosis-related markers, rather than establishing direct functional modulation or suppression of the necroptosis pathway itself.

Several studies have reported the utility of immunohistochemistry for assessing the immunoreactivity and cellular localization of necroptotic pathway proteins in different tissues and disease models. For instance, phosphorylated RIPK3 and MLKL have been detected in neurons, endothelial cells, and CD11b⁺ immune cells in pericontusional brain tissue [42]. Similarly, Li et al. [43] demonstrated that immunohistochemistry provides valuable visual evidence for the spatial distribution and localization of necroptosis-associated markers in specific cell types within testicular tissue.

RIPK1 is likely detectable in normal cerebellar and other brain cells, whereas RIPK3 is typically undetectable unless activated by pathological conditions [44]. RIPK3 expression is usually associated with pathological conditions and is not found in healthy neural tissue [45]. RIPK1 has broader functions than cell death, such as inflammation and cell survival, which may explain its presence in healthy tissue [44].

CCL₄ may be associated with alteration in necroptosis-associated markers through the induction of oxidative stress and TNF-α signaling [46]. This type of cell death contributes to the emergence of several degenerative and inflammatory diseases [47]. Together, MLKL, RIPK1, and RIPK3 form a necrosome complex during necroptosis. Furthermore, RIPK3 phosphorylates MLKL to produce phosphorylated MLKL, which is then transported to the plasma membrane, compromising cell integrity and leading to necroptotic cell death [48]. Activated MLKL leads to osmotic stress, ultimately resulting in cell bursting. The damage-associated molecular pattern (DAMP), which is produced when MLKL-dependent cell death occurs through the necroptotic route, raises inflammation and starts necroptosis and other parallel death pathways [49]. Caspase-8 normally functions as a negative regulator of necroptosis by cleaving RIPK1 and RIPK3, thereby preventing necrosome assembly [50].

Both GFAP and Iba1 are valuable markers for evaluating glial responses in cerebellar pathology, providing insights into astrocyte and microglial activation, respectively [51]. The increased area percentages of GFAP and Iba1 immunoreactivity observed following CCL_4_ administration reflect neuroinflammatory reactivity in the cerebellum. In contrast, the Losartan- and LP-NPs-treated groups showed marked reductions in these markers. While these findings suggest attenuation of glial activation, additional mechanistic studies are required to determine whether these effects result from direct modulation of glial cells or occur secondary to reduced tissue injury and oxidative stress.

These results were consistent with those of Jiménez-Torres et al. [52], which discovered that rats treated with CCL₄ had higher levels of GFAP in their cerebellum. The CCL₄ toxicity-induced increase in GFAP expression in the cerebellum can be explained by oxidative stress in brain tissue, which raises MDA and NO levels and lowers antioxidant defenses (GSH, SOD, and GPX). This oxidative damage can activate astrocytes and increase GFAP production [3]. Additionally, CCL₄ exposure has been associated with a decrease in Purkinje cells in the cerebellum, which may trigger a compensatory response from astrocytes and raise GFAP expression [52].

CCL₄ induces oxidative stress in the brain, which could affect microglial activation and Iba1 expression [53]. Losartan may normalize Iba1 immunoreactivity by reducing oxidative stress brought on by CCL₄ through its ability to lower inflammatory markers. Furthermore, as observed in Iba1 IHC, alterations in microglial morphology or density, as well as its anti-inflammatory properties, may cause microglia to become less reactive. Specific research is necessary to validate these theories and experimentally confirm the actual protective effect.

Purkinje cells count and the area percentage of myelinated nerve fibers both decreased significantly in the CCL₄ group compared to the control group, suggesting that CCL₄ has a neurotoxic effect. Brain tissue morphological changes, swelling, vacuolar degeneration, and karyopyknosis can all result from exposure to CCL₄ [54, 55].

These findings indicate that CCL₄-induced cerebellar injury is associated with alterations in necroptosis-related and oxidative stress markers, while losartan and LP-NPs were associated with restoration of these changes. However, the current work was not designed to demonstrate direct mechanistic involvement of the necroptosis pathway, and further investigations are required to confirm the molecular pathways underlying the observed protective effects.

Moreover, LP-NPs were associated with greater relative attenuation of CCL_4_-induced cerebellar injury in selected histological and immunohistochemical parameters compared to conventional losartan. Although LP-NPs were synthesized as carrier-free drug nanocrystals consisting solely of pure losartan potassium without matrix components, this study did not evaluate nanoparticle pharmacokinetics, brain tissue drug accumulation, or cellular uptake. Consequently, the relative improvements observed with LP-NPs are presented solely as an experimental observation and reported as empirical biological outcomes within this model.

Although the present study demonstrated the biochemical, molecular, and histological protection effects of losartan and LP-NPs against CCL₄-induced cerebellar injury, several limitations should be acknowledged. First, pharmacokinetic evaluation, brain biodistribution analysis, and comprehensive in vivo assessment of nanoparticle behavior were not performed; therefore, the mechanisms governing systemic disposition, tissue targeting, and biological fate of LP-NPs remain to be fully elucidated. Second, the absence of functional and behavioral assessments limits evaluation of cerebellar performance; therefore, future studies incorporating tests of motor coordination are warranted to strengthen the translational relevance of the findings. In addition, while immunohistochemical analysis provided useful spatial localization, semi-quantitative IHC area percentage alone cannot validate protein-level modulation or pathway activity. Because fresh-frozen tissue was unavailable, quantitative protein assays (such as Western blotting or ELISA) could not be performed; therefore, the present IHC findings are interpreted strictly as descriptive, spatial marker alterations rather than quantitative protein estimation. Additionally, direct functional validation of necroptosis signaling requires assessment of specific phosphorylation events, particularly phosphorylated RIPK3 (p-RIPK3) and phosphorylated MLKL (p-MLKL). Additionally, the study lacked the healthy Losartan-alone and sham-milled vehicle control groups. Although LP-NPs are drug-only nanocrystals without carrier excipients, physical changes from top-down milling (e.g., particle size reduction) could influence outcomes independently. Including these controls in future studies will enable clearer discrimination between baseline drug safety, physical formulation effects, and therapeutic efficacy.

Moreover, the physicochemical characterization of the nanocrystal formulation was not comprehensively assessed, as parameters including particle size distribution, polydispersity index (PDI), zeta potential, dissolution behavior, and long-term stability were not evaluated. Consequently, all comparative findings regarding LP-NPs in this study are restricted strictly to observational biological endpoints, and no conclusions can be drawn regarding pharmacokinetic superiority, enhanced bioavailability, or translational applicability. Regarding qRT-PCR methodology, primer amplification efficiencies were not experimentally validated via serial cDNA dilution curves, representing a limitation in strict compliance with MIQE guidelines. A study limitation is the absence of an interim Week 6 sacrifice group to quantify baseline CCL_4_ injury prior to treatment; thus, therapeutic effects are reported as attenuation of persistent toxicity relative to age- and time-matched untreated controls at Week 10.

Furthermore, although the sample size was determined by an a priori power analysis, the relatively small sample size and the use of only male rats in a toxin-induced model may limit the generalizability of the findings and mechanistic interpretation. Future studies with larger cohorts and complementary experimental models are warranted.

## Conclusions

The present study evaluated the effects of Losartan and Losartan-loaded nanoparticles (LP-NPs) on CCL₄-induced cerebellar alterations in male rats. Exposure to CCL₄ induced marked oxidative stress, histological alterations, and altered expression of necroptosis-related markers (upregulated *Tnf-α*, *Ripk1*, *Ripk3*, and *Mlkl* mRNA expression, along with altered immunoreactivity of RIPK1, RIPK3, and caspase-8). Administration of Losartan and LP-NPs was associated with attenuated oxidative stress and improved tissue morphology, with LP-NPs showing marked improvement in oxidative stress biomarkers, histological parameters, and selected IHC markers. Both treatments produced comparable reductions in qRT-PCR mRNA expression levels of *Tnf-*α*, Ripk1*, and *Ripk3*.

Importantly, because activated or phosphorylated pathway proteins (e.g., p-RIPK3, p-MLKL) were not assessed, these findings indicate an association with necroptosis-related molecular changes rather than proven activation or suppression of the necroptosis pathway. Furthermore, as these observations are limited to a preclinical male rat model, further mechanistic and functional studies including both sexes are warranted and necessary to determine any broader biological or translational relevance.

## Supplementary Information

Below is the link to the electronic supplementary material.

Supplementary file1 (DOCX 277 KB)

## Data Availability

On request, data will be made available.
