# Supplementary material for: Impact of Losartan Nanoparticles on Carbon Tetrachloride-induced Cerebellar Injury in Rats: Association with Alterations in RIPK1/RIPK3/MLKL Necroptosis Markers
Source: Mol Neurobiol. 2026 Sep 17;63(1):901. doi: 10.1007/s12035-026-06178-z (PMC13582310; doi:10.1007/s12035-026-06178-z)
Supplement: Supplementary file 1 [file 12035_2026_6178_MOESM1_ESM.docx]

| A) **Gapdh** | |
| --- | --- |
|  |  |
| B) **Tnf alpha** | |
|  |  |
| C) **Ripk1** | |
|  |  |
| D) **Ripk3** | |
|  |  |
| E) **Mlkl** | |
|  |  |

**Supplementary Figure S1: Representative amplification plots and melting curves for *Gapdh*, *Tnf-α*, *Ripk1*, *Ripk3*, and *Mlkl*. All targets showed characteristic sigmoidal amplification curves and a single sharp melting peak, indicating specific amplification without detectable primer-dimer formation.**
